# Newly Diagnosed Graves' Disease Followed by the Discovery of a Cavernous Internal Carotid Artery Aneurysm Causing Panhypopituitarism: A Case Report

**DOI:** 10.7759/cureus.116662

**Published:** 2026-09-21

**Authors:** Ibrahim M Touffaha, Georges Elhomsy

**Affiliations:** 1 Medical Education, University of Kansas School of Medicine-Wichita, Wichita, USA; 2 Endocrinology and Diabetes, University of Kansas Medical Center, Wichita, USA

**Keywords:** graves’ disease, hyperprolactinemia, hypopituitarism, intracranial aneurysm growth, pituitary gland

## Abstract

Sellar and parasellar vascular lesions are rare causes of pituitary dysfunction and may mimic pituitary adenomas or other sellar masses. Cavernous internal carotid artery (ICA) aneurysms can cause hypopituitarism through compression of the pituitary gland or stalk, but diagnosis may be delayed when symptoms are initially attributed to a more common endocrine disorder.

We report a 37-year-old man who presented with six weeks of unintentional weight loss, fatigue, heat intolerance, excessive sweating, anxiety, tremor, and tachycardia. Initial evaluation showed suppressed thyroid-stimulating hormone (TSH), elevated total triiodothyronine (T3), positive thyrotropin receptor antibody, positive thyroid-stimulating immunoglobulin, and a hypervascular thyroid gland on ultrasound, consistent with Graves’ disease. He was started on methimazole and atenolol. Although thyroid function tests improved, he developed persistent neck discomfort, prompting contrast-enhanced computed tomography (CT), which revealed an enhancing sellar mass. Magnetic resonance imaging (MRI) showed a T2-hypointense sellar lesion with internal flow artifact, and CT angiography confirmed a saccular aneurysm of the cavernous segment of the right ICA. Pituitary hormone evaluation demonstrated secondary adrenal insufficiency, central hypothyroidism, hypogonadotropic hypogonadism, growth hormone (GH) deficiency, and mild hyperprolactinemia, consistent with panhypopituitarism from pituitary compression. The patient was started on prednisone and levothyroxine, and atenolol and prednisone were subsequently discontinued before endovascular embolization was performed. At follow-up, symptoms improved, but panhypopituitarism persisted.

This case highlights the importance of reassessing patients when endocrine symptoms evolve despite an initially appropriate diagnosis.

## Introduction

Sellar and parasellar lesions are an uncommon cause of endocrine disease. The sella is a bony structure that holds the pituitary. Internal carotid artery (ICA) aneurysms may extend toward the sella but rarely cause pituitary dysfunction. Cavernous ICA aneurysms account for 2% of all intracranial aneurysms [1,2] and represent 0.17% of documented hypopituitarism cases [3].

Aneurysm-related pituitary dysfunction involves direct compression of the pituitary gland or stalk and possible compromise of the hypophyseal blood flow [4]. This pituitary dysfunction may lead to partial hypopituitarism, panhypopituitarism, or mild hyperprolactinemia due to the stalk effect [5].

We report a patient who was initially diagnosed with Graves’ disease and was soon after found to have panhypopituitarism due to ICA aneurysm compression of the pituitary gland. This case is important because it highlights how a common endocrine disorder can mask a rare, life-threatening condition, and it emphasizes the need for vigilance when the clinical course is atypical.

## Case presentation

A 37-year-old male patient presented to the endocrinology clinic with a six-week history of unintentional 20-pound weight loss, fatigue, heat intolerance, excessive sweating, and anxiety. Physical examination was notable for mild thyroid enlargement, fine tremors, and tachycardia. Past medical history was unremarkable. Family history was notable for a mother with Hashimoto’s thyroiditis and a daughter with vitiligo. Review of systems was negative, and all cranial nerves were intact.

Initial laboratory workup showed a suppressed thyroid-stimulating hormone (TSH) level of 0.009 µIU/mL, free thyroxine (free T4) of 1.28 ng/dL, total triiodothyronine (T3) of 226 ng/dL, thyrotropin receptor antibody of 4.66 IU/L, and thyroid-stimulating immunoglobulin of 2.24 IU/L. Thyroid ultrasound revealed an enlarged, heterogeneous, and hypervascular thyroid gland with no discrete nodules. These findings are consistent with Graves’ disease, and the patient was started on methimazole 10 mg and atenolol 25 mg. Table 1 shows serial lab values with normal lab values. Serial thyroid function test trends over time are presented in Figure 1.

**Table 1:** Serial endocrine laboratory trends Note: Reference ranges are shown in the column headers. “-” indicates that the laboratory test was not obtained at that time point. Total T3 and free T3 are reported separately because they use different assays and units. Normal values are in parentheses in the header. Abbreviations: ACTH, adrenocorticotropic hormone; FSH, follicle-stimulating hormone; IGF-1, insulin-like growth factor 1; LH, luteinizing hormone; TRAb, thyrotropin receptor antibody; TSH, thyroid-stimulating hormone; TSI, thyroid-stimulating immunoglobulin.

Time point	Clinical context	TSH, µIU/mL (0.450-4.500)	Free T4, ng/dL (0.82-1.77)	Total T3, ng/dL (71-180)	Free T3, pg/mL (2.0-4.4)	TRAb, IU/L ( 0.00-1.75)	TSI, IU/L (0.00-0.55)	ACTH, pg/mL (7.2-63.3)	AM cortisol, µg/dL (6.2-19.4)	LH, mIU/mL (1.7-8.6)	FSH, mIU/mL (1.5-12.4)	Total testosterone, ng/dL (264-916)	Free testosterone, pg/mL (8.7-25.1)	IGF-1, ng/mL (90-278)	Prolactin, ng/mL (3.9-22.7)
On Presentation															
Initial visit	On presentation & methimazole 30 mg daily started	0.009	1.28	226	-	4.66	2.24	-	-	-	-	-	-	-	-
4 weeks after presentation	Methimazole lowered to 20 mg daily	0.016	0.89	177	-	-	-	-	-	-	-	-	-	-	-
8 weeks after presentation	Methimazole lowered to 10 mg daily	0.814	0.45	104	-	-	-	-	-	-	-	-	-	-	-
12 weeks after presentation	Methimazole lowered to 5 mg daily	0.798	0.58	-	2.8	-	-	-	-	-	-	-	-	-	-
16 weeks after presentation	Methimazole was stopped	2.58	0.57	-	2.4	-	-	-	-	-	-	-	-	-	-
20 weeks after presentation	ICA aneurysm was noted and levothyroxine 25 mcg and prednisone 5 mg were started	0.99	0.8	-	-	-	-	7.3	1.7	-	-	-	-	76	30.1
28 weeks after presentation	4 weeks after coil embolization, prednisone taper started, and TRT started	0.535	0.9	-	1.9	-	-	-	-	0.6	2	<3	<0.2	113	20.9
38 weeks after presentation	2 weeks after stopping prednisone	0.05	1.03	-	-	-	-	7.7	1	-	-	475	-	-	-
1 year since presentation	Patient remains on prednisone, levothyroxine, and TRT	1.02	1.31	-	2.9	-	-	12.4	5.7	-	-	941	-	-	-

**Figure 1:**
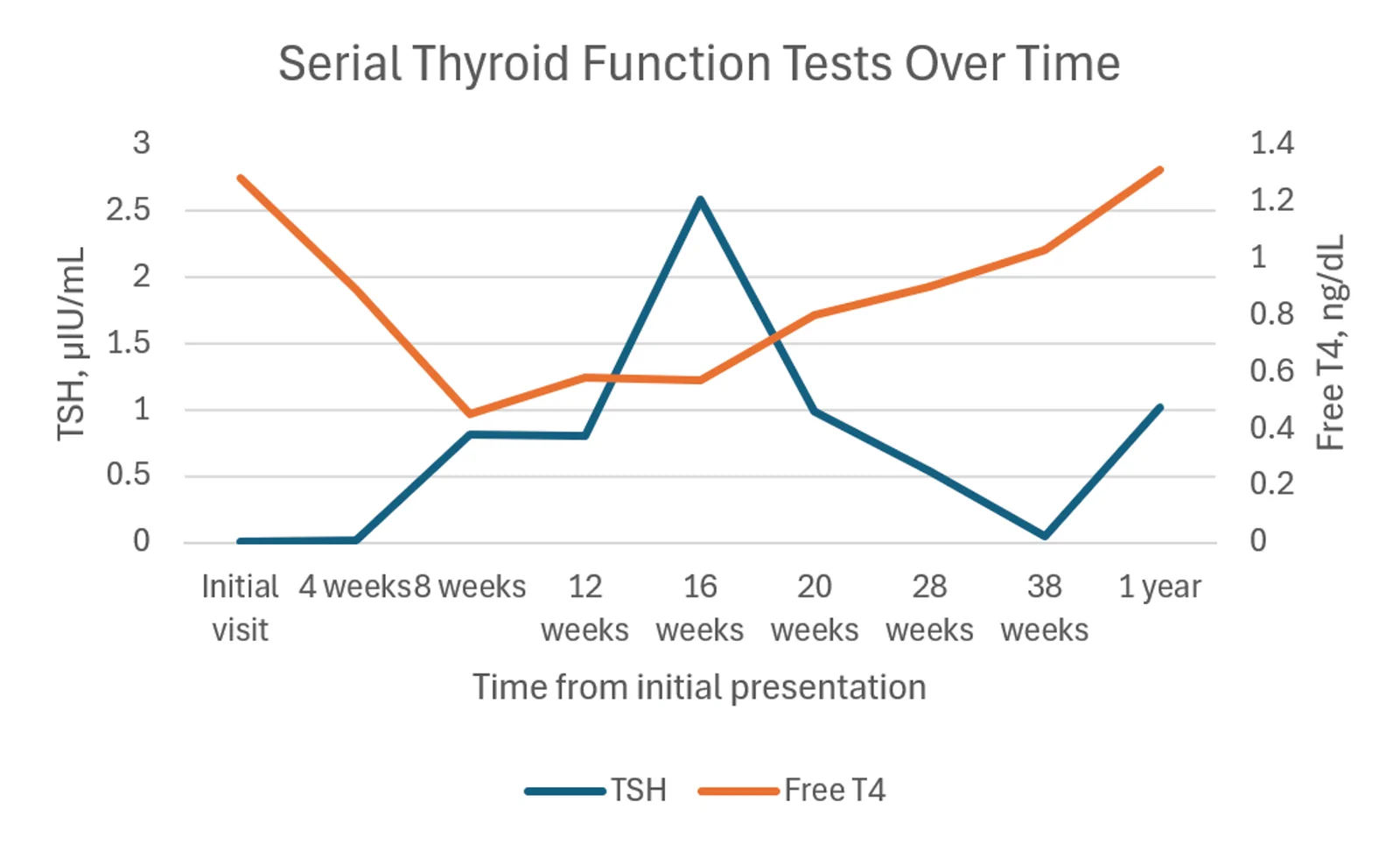
Serial thyroid function test trends over time Thyroid-stimulating hormone (TSH) and free thyroxine (T4) values are shown from initial presentation through one-year follow-up. Exact numerical values and additional endocrine laboratory results are provided in Table 1.

At the follow-up visit, thyroid function tests improved; however, the patient began to report generalized nonspecific neck discomfort. Contrast-enhanced computed tomography (CT) of the neck was obtained to evaluate the soft tissues. The CT incidentally demonstrated an enhancing sellar mass measuring approximately 2.2 cm.

Magnetic resonance imaging (MRI) of the brain with and without contrast showed a T2-hypointense sellar mass with an internal flow artifact suggesting a vascular lesion. CT angiography of the head and neck confirmed a saccular aneurysm arising from the mid-to-distal cavernous segment of the right ICA, measuring 2.5 × 2.2 × 2.1 cm. Figure 2 shows the CT angiogram.

**Figure 2:**
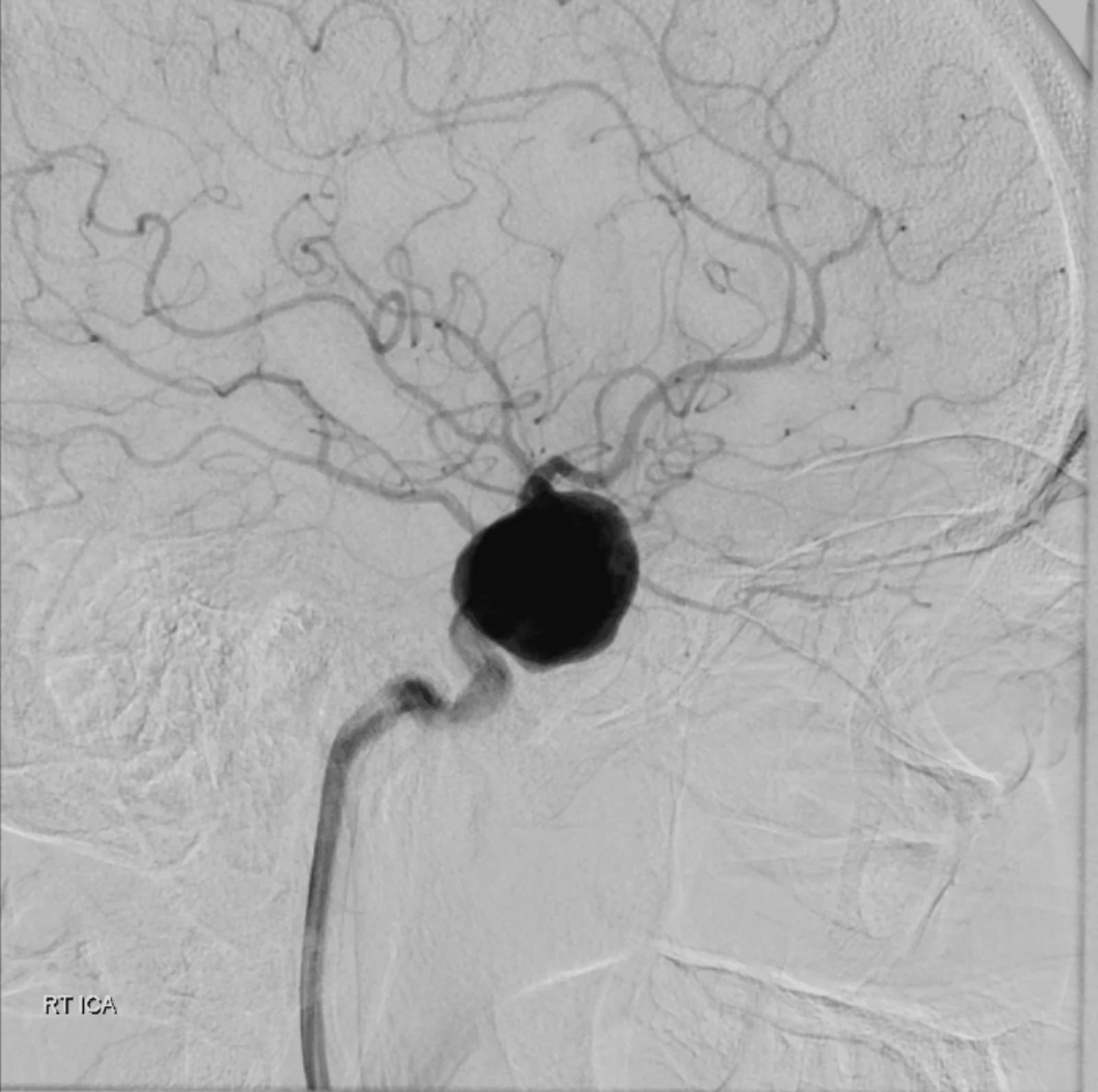
Pre-embolization right internal carotid artery angiogram demonstrating a large, cavernous internal carotid artery aneurysm

Given the concern for pituitary compression by the aneurysm, a complete pituitary hormone evaluation was obtained. Results showed adrenocorticotropic hormone (ACTH) 7.3 pg/mL, 8 AM cortisol 1.7 µg/dL, TSH 0.99 µIU/mL, free T4 0.8 ng/dL, luteinizing hormone (LH) 0.6 mIU/mL, follicle-stimulating hormone (FSH) 2.0 mIU/mL, total testosterone <3 ng/dL, insulin-like growth factor 1 (IGF-1) 76 ng/mL, and prolactin 30.1 ng/mL.

The laboratory findings were consistent with panhypopituitarism secondary to aneurysmal compression of the pituitary gland. Specifically, the patient had secondary adrenal insufficiency, secondary hypothyroidism, growth hormone (GH) deficiency, hypogonadotropic hypogonadism, and hyperprolactinemia due to pituitary stalk compression.

The patient was started on prednisone for the adrenal insufficiency and levothyroxine 25 µg daily for hypothyroidism. Atenolol and methimazole were discontinued, lisinopril 5 mg daily was initiated, and endovascular embolization was planned.

The patient underwent embolization of the aneurysm and was followed up in the clinic. A CT angiogram post-embolization is shown in Figure 3. At follow-up, the patient reported symptomatic improvement; however, his panhypopituitarism persisted, but the weight loss and tachycardia had resolved. 

**Figure 3:**
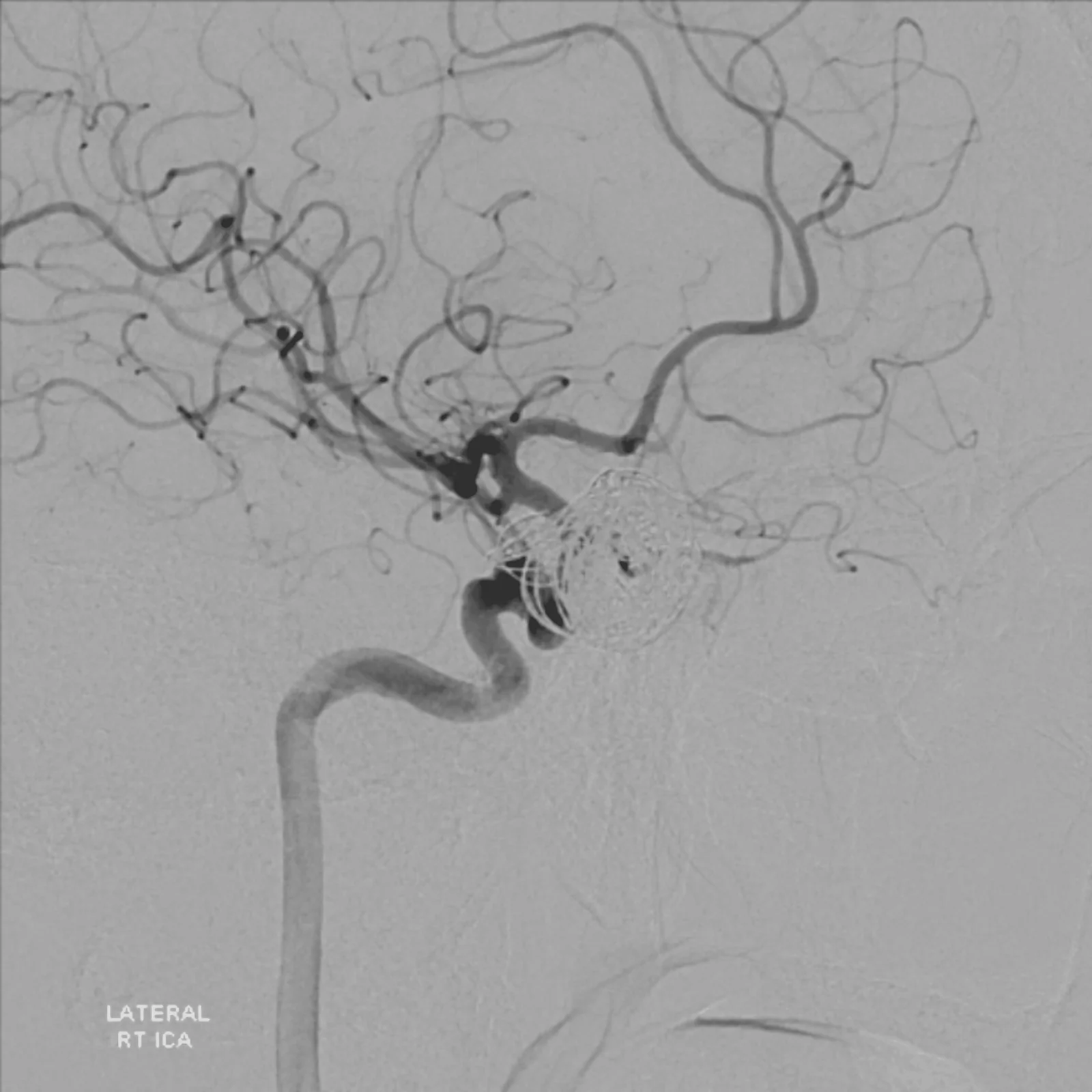
Post-treatment digital subtraction angiography of the right internal carotid artery demonstrating endovascular embolization of the cavernous internal carotid artery aneurysm

## Discussion

This case describes a patient who initially presented with laboratory findings and symptoms consistent with Graves’ disease but was subsequently found to have an ICA aneurysm compressing the pituitary gland and causing panhypopituitarism. The case highlights how a common endocrine disorder can mask or delay recognition of a second, unrelated, and potentially life-threatening condition.

The clinical presentation of ICA aneurysms varies, ranging from headaches and visual deficits to cranial neuropathies [5] or, as in this case, isolated endocrine dysfunction with mild neck discomfort. The mechanism of pituitary failure is thought to involve direct mechanical compression of the gland and stalk, with possible compromise of the superior hypophyseal arteries, leading to ischemia [6] and anterior pituitary hormone deficiencies, particularly ACTH, TSH, and gonadotropins, with mild hyperprolactinemia secondary to the stalk effect.

The patient’s concurrent Graves’ disease contributed to the diagnostic challenge. Symptoms such as weight loss, heat intolerance, and fatigue were attributed to hyperthyroidism, while overlapping features of secondary adrenal insufficiency and hypogonadism may have been overlooked. This underscores the importance of maintaining a broad differential diagnosis in patients with evolving or unexplained endocrine symptoms, even when a primary disorder has been identified. Although there is no established causal link between autoimmune thyroid disease and intracranial aneurysm formation, coincidence is most likely in this case.

Management requires both endocrine stabilization and definitive treatment of the vascular lesion. Prompt initiation of glucocorticoids is essential in patients with secondary adrenal insufficiency before addressing hyperthyroidism, as uncontrolled thyrotoxicosis can increase cortisol clearance and precipitate adrenal crisis [7]. Definitive management of ICA aneurysms may involve microsurgical clipping or, more commonly in recent years, endovascular approaches such as coil embolization or flow-diverting stent placement [8-10]. Prognosis for pituitary function recovery is variable; in published series, many patients require lifelong hormone replacement, though partial recovery has been documented, particularly when intervention occurs before irreversible gland damage [9].

This case reinforces that clinicians should consider additional evaluation when the clinical picture is atypical or when multiple anterior pituitary hormone deficits are present. Multidisciplinary coordination between endocrinology, neurosurgery, and radiology is essential to ensure accurate diagnosis and optimal patient outcomes.

## Conclusions

This case highlights an unusual diagnostic sequence in which newly diagnosed Graves’ disease was followed by the discovery of a cavernous ICA aneurysm causing panhypopituitarism. Although the patient’s initial thyrotoxic symptoms were appropriately attributed to Graves’ disease, persistent symptoms and evolving laboratory findings prompted further evaluation and revealed a second, clinically significant process. Clinicians should maintain a broad differential diagnosis when endocrine symptoms do not follow the expected course, especially when laboratory findings suggest central pituitary dysfunction. Early recognition of aneurysm-related pituitary compression is important to guide hormone replacement, prevent adrenal crisis, and coordinate timely multidisciplinary management.
